# Supplementary material for: Heterologous DNA–Adenovirus Prime–Boost Strategy Expressing Bluetongue Virus VP2 and VP7 Proteins Protects Against Virulent Challenge
Source: Vaccines (Basel). 2025 Sep 22;13(9):991. doi: 10.3390/vaccines13090991 (PMC12474434; doi:10.3390/vaccines13090991)
Supplement: Supplementary file 1 [file vaccines-13-00991-s001.zip › Table S1.pdf]

Table S1. Clinical score evaluation in BTV-infected IFNAR$^{(-/-)}$ mice.

Observation	Score
**Body weight**	
- No change	0
- Loss of body weight in % = score points (e.g. loss of 8% body weight = 8 points)	1-25
- Loss of >25%	25
**Appearance**	
Fur	
- Shinning	0
- Matte	2
- Ruffled	5
Eyes	
- Clear	0
- Unclean, sticky, closed, semi-closed	5
Posture	
- Normal	0
- Hunched	5
- Massively hunched	15
**Motility**	
- Spontaneous (normal behaviour + social contact)	0
- Spontaneous but reduced	1
- Moderately reduced	5
- Motility only after stimulation	15
- Coordination disorder	20
- Lethargy	25
**Respiration**	
- Breathing normal	0
- Breathing slightly changed	1
- Accelerated breathing (>30%)	10
- Strongly accelerated breathing (>50%)	25
**Severity rating**	**Sum score**
Severity level 0: No burden, animals are healthy	0-5
Severity level 1: Low disease burden	5-10
Severity level 2: Moderate disease burden	10-25
Severity level 3: Moderate to severe sickness	25-50
Severity level 4: Severe burden (Implement human endpoint)	>50
