# Supplementary figures and images for: Heterologous DNA–Adenovirus Prime–Boost Strategy Expressing Bluetongue Virus VP2 and VP7 Proteins Protects Against Virulent Challenge
Source: Vaccines (Basel). 2025 Sep 22;13(9):991. doi: 10.3390/vaccines13090991 (PMC12474434; doi:10.3390/vaccines13090991)

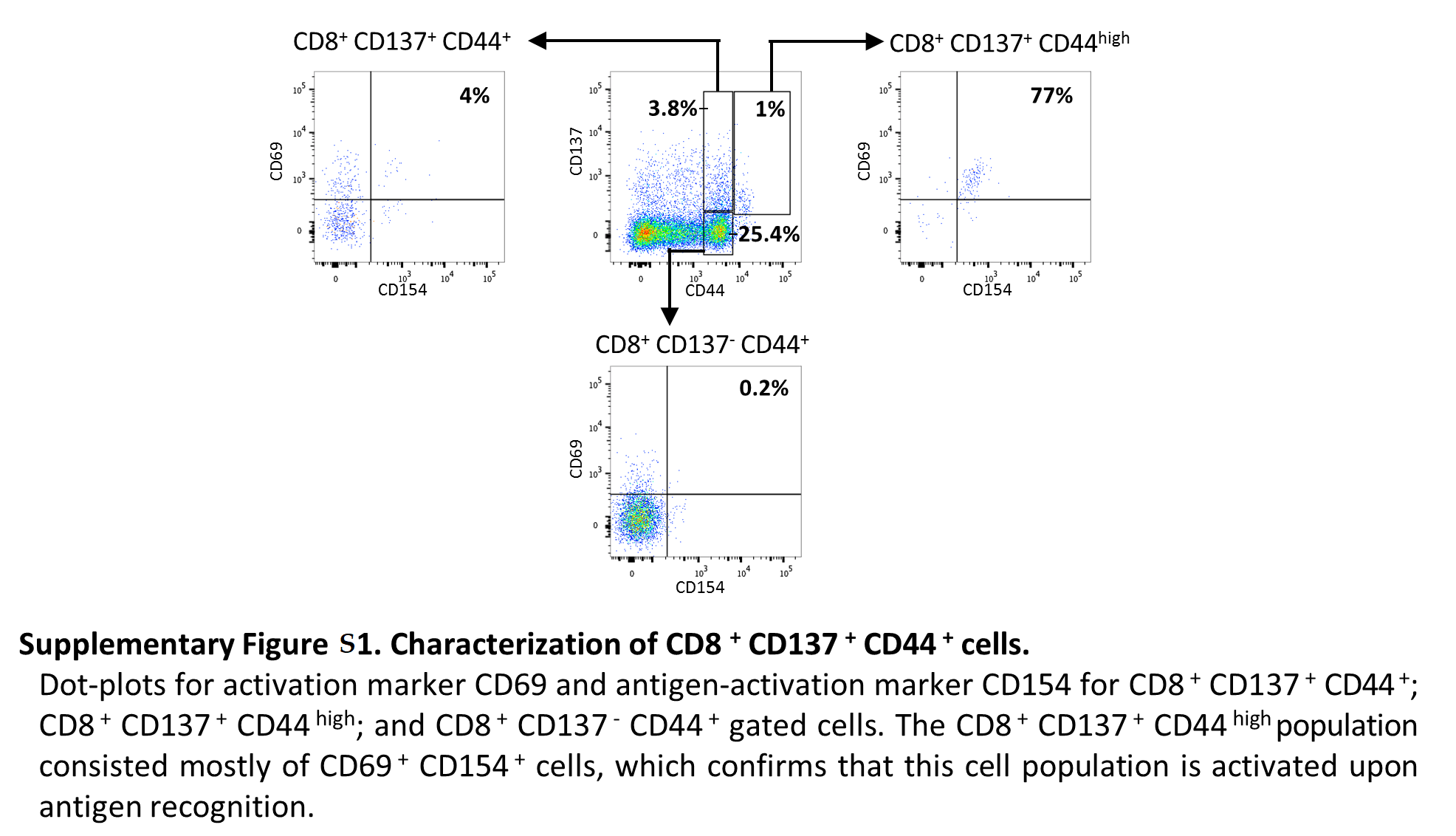

Supplement: Supplementary file 1 [file vaccines-13-00991-s001.zip › Supplementary Figure 1-Characterization of CD8+ CD137+ CD44++ cells.tif]
